# Loss of mitochondrial DNA is associated with reduced DNA content variability in *Saccharomyces cerevisiae*

**DOI:** 10.17912/micropub.biology.001117

**Published:** 2024-03-11

**Authors:** Christopher D. Putnam

**Affiliations:** 1 Department of Medicine, University of California, San Diego, San Diego, California, United States

## Abstract

DNA content measurement by fluorescence-assisted cell sorting (FACS) provides information on cell cycle progression and DNA content variability. *Saccharomyces cerevisiae* mutants with DNA content variability that was reduced relative to wild-type strains had defects in mitochondrial DNA (mtDNA) maintenance and mitochondrial gene expression and were correlated with strains found to lack mtDNA ([ *rho ^0^* ] cells) by genome sequencing and fluorescence microscopy. In contrast, mutants with increased variability had defects in cell cycle progression, which may indicate a loss of coordination between mtDNA and nuclear DNA replication. Thus, FACS measurement of DNA content variability can provide insight into cell-to-cell heterogeneity in mtDNA copy number.

**Figure 1. DNA content variability as observed by FACS in S. cerevisiae is caused by heterogeneity in mtDNA copy number:**
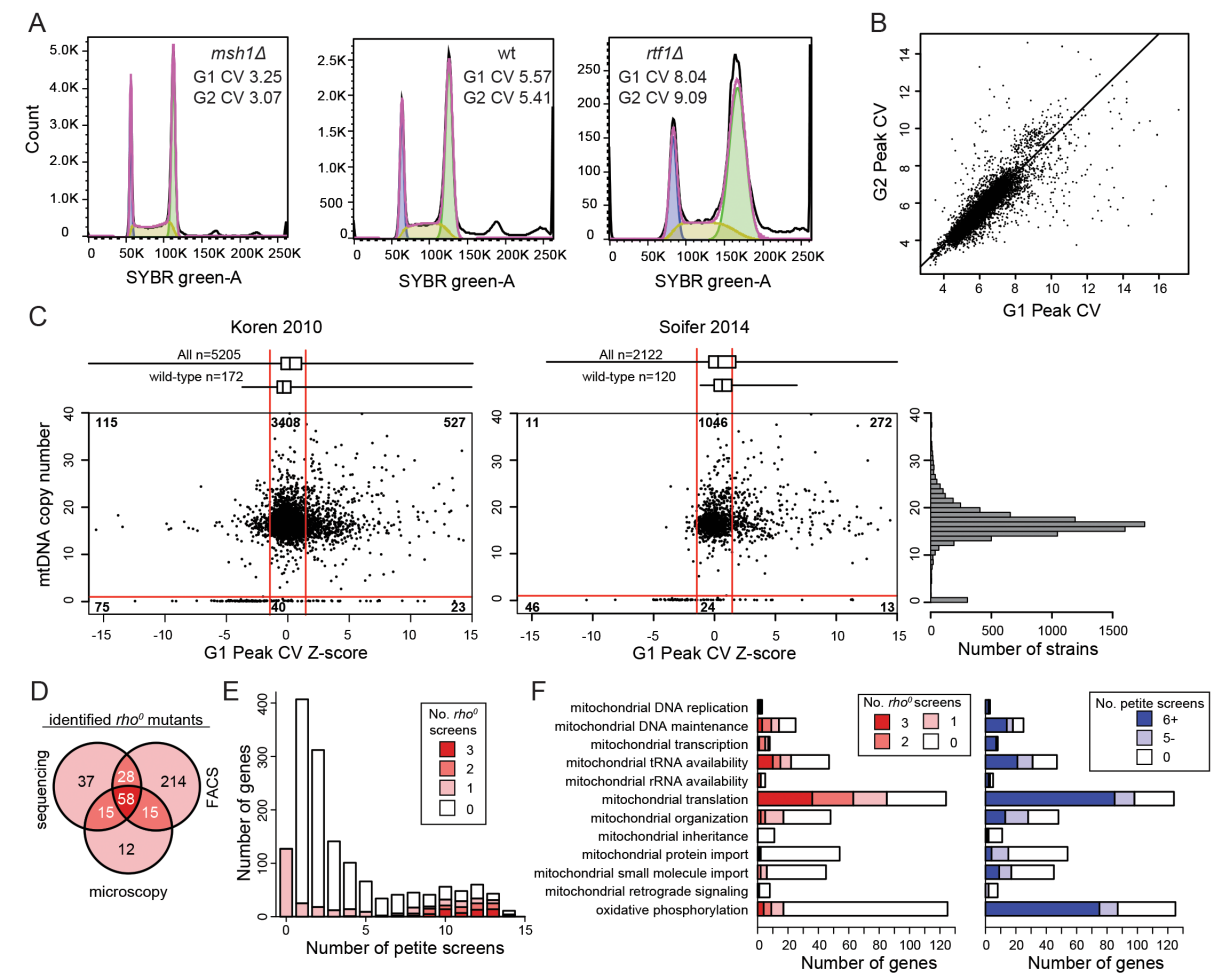
**A.** DNA content measurements from log-phase cells for example strains with low ( *msh1Δ* ), normal (wild-type), and increased ( *rtf1Δ* ) Peak CV values determined using FlowJo. The fitted cell cycle distribution (purple line) shows the cells assigned to the G1 phase (blue), S phase (yellow) and G2 phase (green). **B.** The G1 Peak CV values and G2 Peak CV values are highly correlated, consistent with having a similar underlying cause. Plotted data include both the Koren 2010 and Soifer 2014 data sets. **C.** Comparison of mutant strains with significantly reduced G1 Peak CV Z-scores in the Koren 2010 data set (left) and the Soifer 2014 data set (right) with the estimated mtDNA copy number from genome sequencing. Box-and-whisker plots for all G1 Peak CV Z-scores and for the wild-type G1 Peak CV Z-scores are shown at top. Significance thresholds (Z-scores of ±1.5, vertical red lines) for the G1 Peak CV Z-scores and for sequence estimates of mtDNA content (1 copy per cell, horizontal red line) are shown. Numbers in the scatter plot indicate the number of mutant strains that fall into each region of the graph. The histogram of mutants with different estimated mitochondrial DNA copy numbers is shown on right. **D.** Overlap of genes associated with [ *rho ^0^* ] mutants identified by FACS, genomic sequencing, and fluorescence microscopy. **E.** Histogram of the number of genes identified by different numbers of screens in 14 different screens for petite mutations. Colored bars indicate genes that have been identified in 1, 2, or 3 [ *rho ^0^* ] screens (Panel D), demonstrating that [ *rho ^0^* ] mutations are enriched in those mutations identified in 7 or more petite screens. **F.** The number of genes in each mitochondrial functional category colored based on the number of [ *rho ^0^* ] screens the gene was identified in (left, red) or colored based on the number of petite screens the gene was identified in (blue, right).

## Description

DNA content measurements by fluorescence-assisted cell sorting (FACS) of fixed and stained *Saccharomyces cerevisiae* strains are useful to determine the distribution of cells in the cell cycle both for synchronized and log-phase cells. Reanalysis of previously published FACS data that measured DNA content of log-phase cells from the haploid deletion collection strains (Extended Data; (Koren, Soifer, & Barkai, 2010; Soifer & Barkai, 2014) ) revealed that the width of the fitted G1 and G2 peaks varied widely, indicating differing levels of DNA content heterogeneity within cultures ( Figure 1A ). For example, the strain with a deletion of *MSH1* , which encodes the MutS homolog involved in mitochondrial DNA (mtDNA) repair and maintenance (Reenan & Kolodner, 1992) , was observed to have narrower G1 and G2 peaks and thus less DNA content heterogeneity than the wild-type strain ( Figure 1A ). In contrast, the strain with a deletion of *RTF1* , which encodes a subunit of the Paf1 transcription elongation complex (Mueller & Jaehning, 2002) , was an example of a strain observed to have wider G1 and G2 peaks and thus more DNA content heterogeneity ( Figure 1A ). The coefficients of variation (CV) of the G1 and G2 peaks, which are the standard deviation of the fitted peaks normalized by their means and provide a means to quantitate this heterogeneity, were strongly correlated (Pearson coefficient 0.83, Spearman coefficient 0.89; Figure 1B ), indicating that the DNA content heterogeneity was generally not cell cycle dependent.

Many mutants with low G1 Peak CV values had defects in mitochondrial functions, suggesting the hypothesis that reduced DNA content variability is associated with loss of mitochondrial DNA (mtDNA). Unlike many other eukaryotes, *S. cerevisiae* can survive without mitochondrial function, although loss of the mitochondria themselves is lethal (McConnell, Stewart, Talin, & Yaffe, 1990) . Cells without mitochondrial function are termed ‘petite’, as they have reduced colony size due to reduced synthesis of the amino acids glutamate, glutamine, arginine and leucine (Ephrussi, Hottinguer, & Tavlitzki, 1949; Vowinckel et al., 2021) . Petite strains are caused by many defects (Contamine & Picard, 2000; Tzagoloff & Dieckmann, 1990) : (1) mutations in nuclear genes required for growth on non-fermentable carbon sources, (2) mutations in mitochondrial genes required for growth on non-fermentable carbon sources, and (3) partial deletions ([ *rho ^-^* ]) or complete loss ([ *rho ^0^* ]) of mtDNA, which can be due to mutations in genes required for mtDNA replication and maintenance (e.g. *MIP1* , *MSH1* , and *PIF1* ).

To systematically test the hypothesis that lowered DNA variability was linked to loss of mtDNA, the estimates of the mtDNA copy number in different mutant based on genome sequencing of strains from the yeast deletion collection (average 16 ± 2 copies per cell; (Puddu et al., 2019) ) were compared with the G1 Peak CV values from FACS analysis ( Figure 1C ). The G1 Peak CV values were affected by batch effects that could be observed when comparing the Koren 2010 data set, which included the entire yeast deletion collection, and Soifer 2014 data set, which included only a subset of the deletion collection. Moreover, batch effects were observed between the individual experiments in each FACS data set. This variability is likely due to subtle differences in growth conditions, as the mtDNA copy number change during growth (Galeota-Sprung, Fernandez, & Sniegowski, 2022) . To accommodate this variability, modified z-scores for the G1 Peak CV values were calculated for each experiment after removing outliers, and cutoffs for strains with significantly altered G1 Peak CV values were placed at modified z-scores of -1.5 and +1.5 ( Figure 1C ).

Genome sequencing identified 144 [ *rho ^0^* ] mutants of 138 genes (Puddu et al., 2019) , using a definition of <1 copy of mtDNA per cell ( Figure 1C ). In both FACS data sets, strains with significantly reduced G1 Peak CV values were highly enriched for these sequencing-identified [ *rho ^0^* ] mutants (p<2x10 ^-16^ for both FACS data sets; Fisher’s exact test; Figure 1C ). Mutant strains identified by all three screens included those with deletions of genes with known mtDNA maintenance functions (such as *MIP1* and *PIF1* ), as well as deletions of genes involved in mitochondrial protein translation (such as *MRPL35* and *RMD9* ). Of the 66 mutants identified with low G1 Peak CV values in both FACS data sets, 47 were also identified as [ *rho ^0^* ] mutants by genome sequencing. The 19 mutants not identified by sequencing affected genes that were also associated with mitochondrial translation ( *MRP17* , *MRPL3* , *MRPL15* , *MRPL31,* *MRPL36* , *MRPS5* , *MRX14* , *MST1* , *MTG1* , and *RSM26* ) and other mitochondrial functions ( *ATP4* , *HEM14* , *INH1* , *ISA1* , and *PPT2* ) as well as other functions that were less obviously related to mitochondria ( *AGP2* , *FDO1* , *NRP1* , and *YHR175W-A* ). Remarkably, loss of *ATP4* , which encodes an F _0_ F _1_ -ATPase subunit, has been previously linked to rapid loss of mtDNA (Merz & Westermann, 2009; Paul, Velours, Arselin de Chateaubodeau, Aigle, & Guerin, 1989) . The [ *rho ^0^* ] mutants identified by sequencing and by FACS also overlapped well with those identified by fluorescence microscopy of DAPI-stained respiratory-deficient strains ( Figure 1D ) (Zhang & Singh, 2014) . Taken together, these data indicate that significantly low DNA content variability as measured by FACS is associated with mtDNA loss.

The imperfect overlap of hits from different screens of the yeast deletion collection is a well-known phenomenon (see for instance (Putnam et al., 2016) ). The arrayed deletions are subject to the accumulation of suppressor mutations, chromosomal rearrangements, and aneuploids (Puddu et al., 2019) as well as cross-contamination of mutant strains grown on the same plate that can lead to both false positive and false negative results (Putnam et al., 2016) . These challenges are typically managed by analyzing the results by focusing on implicated pathways instead of identified genes. Additionally, [ *rho ^0^* ] formation may be further complicated by its underlying biology. In the case of *atp16Δ* mutants, mtDNA defects due to both loss and internal deletions (e.g. both [ *rho ^0^* ] and [ *rho ^-^* ] mutants) accumulate due to selective pressure, as mtDNA defects prevent passive proton transport through the F _0_ sector in Atp16-depleted cells (Duvezin-Caubet et al., 2006) . Thus, the lack of concordance between the different assays for [ *rho ^0^* ] mutants can also be due to mutations that become petite through generation of either [ *rho ^0^* ] cells, which would be identified in these assays, or [ *rho ^-^* ] cells, which would not. Moreover, strains without mtDNA maintenance defects can also spontaneously generate [ *rho ^0^* ] isolates during the deletion library propagation due to the high rate of mtDNA instability in the *S. cerevisiae* S288c strain background (Dimitrov, Brem, Kruglyak, & Gottschling, 2009) . Consistent with this, the genome sequencing of multiple isolates of individual deletion strains revealed both [ *rho ^0^* ] and [ *rho ^+^* ] isolates for 17 different mutations (Puddu et al., 2019) .

Most [ *rho ^0^* ] mutants identified by multiple techniques were identified in over half of 14 screens for petite mutations ( Figure 1E ; (Acton et al., 2017; Dimmer et al., 2002; Dudley, Janse, Tanay, Shamir, & Church, 2005; Galardini et al., 2019; Luban, Beutel, Stahl, & Schmidt, 2005; Merz & Westermann, 2009; Qian, Ma, Xiao, Wang, & Zhang, 2012; Steinmetz et al., 2002; Stenger, Le, Klecker, & Westermann, 2020; Tzagoloff & Dieckmann, 1990) ). Not all petite strains are [ *rho ^0^* ], however, due to the multiple mechanisms leading to loss of aerobic respiration (Contamine & Picard, 2000; Tzagoloff & Dieckmann, 1990) . The [ *rho ^0^* ] mutations were enriched for those affecting genes involved in mtDNA maintenance and mitochondrial transcription and translation ( Figure 1F ), whereas defects in oxidative phosphorylation and mitochondrial organization and import were additionally enriched for in mutations only causing a petite phenotype. The requirement for mitochondrial gene expression for retention of mtDNA is well known but remains poorly understood, as mtDNA does not encode genes involved in its maintenance (Myers, Pape, & Tzagoloff, 1985) .

In contrast to the [ *rho ^0^* ] mutants, mutants causing increased G1 Peak CV values are associated with a wide variety of cellular functions, including metabolism, vacuolar function, transcription, and nuclear DNA replication. Of the 1,001 genes implicated by the two data sets, only 167 are in common (Koren et al., 2010; Soifer & Barkai, 2014) , suggesting that many increased G1 Peak CV values may arise from technical considerations. One tendency of mutants with increased G1 Peak CV values is that they tend to come from strains with distributions of log phase cells that differ from the control (p-value < 2.2x10 ^-16^ ; Kolmogorov Smirnov test), which has a percentage of G1, S, and G2 cells of 21.5%, 25.1%, and 53.4% (n=292). This tendency is somewhat stronger in mutants identified in both data sets. These mutant strains are not restricted to those that accumulate in a specific cell cycle phase, and mutants with increased G1, S or G2 can have increased G1 Peak CV values. If the variability in mutant strains with high G1 Peak CV values are due, at least in part, to heterogeneity in mtDNA content, this would suggest that cells that have cell cycle progression defects may disrupt the coordination of mtDNA replication with the nuclear cell cycle. Measuring increased DNA content variability by genome sequencing is complicated by the fact that this analysis averages the content across the sample. Despite this, cells with increased mtDNA content might be expected to have higher DNA content variability; of the 330 mutants with increased mtDNA content by sequencing (p<0.0001; >22.8 copies per cell), only 45 overlap with mutants with increased content variability observed in the Koren and Soifer data sets.

Taken together, these data provide strong support for loss of mtDNA causing the lowered variability of the G1 and G2 peaks observed during FACS analysis of DNA content. This result also indicates that much of the observed variability of DNA content in wild-type cells is due to heterogeneity in mtDNA copy number. Moreover, since FACS analyzes DNA content on a cell-by-cell basis, this analysis suggests that FACS will be useful to study mutations that alter the coordination of mtDNA replication with the nuclear cell cycle and give rise to populations with increased variability of mtDNA copy number between individual cells. An important caveat to the use of this method is that the batch effects observed here indicate that appropriate controls grown under identical conditions must be included in every experiment.

## Methods

FACS data from log phase cells from the *MAT* **a** BY4741 haploid deletion collection (Koren et al., 2010; Soifer & Barkai, 2014) were downloaded from the FlowRepository (Spidlen, Breuer, Rosenberg, Kotecha, & Brinkman, 2012) and were analyzed using the Denn-Jett-Fox model in FlowJo version 10 (FlowJo, 2019) using the Dean-Jett-Fox cell cycle model (Fox, 1980) after removing doublets that ran perpendicular to the laser by gating on a forward scatter height (FSC-H) vs. forward scatter width (FSC-A) plot. Note that cell-cell doublets that are parallel to the laser cannot be excluded by this gate.

## Extended Data

Description: Cell cycle distribution data for strains from the Saccharomyces cerevisiae haploid deletion collection.. Resource Type: Dataset. DOI: 10.22002/v208k-qmb68
